# Prediction of acute mountain sickness risk by pre-plateau uric acid and albumin levels in Chinese young males: a prospective cohort study

**DOI:** 10.7717/peerj.21770

**Published:** 2026-10-14

**Authors:** Hong chen Xie, Xiang qiong Meng, Shen wei Xie, Ze jun Wang, Qi Cui, Xiao ling Tan

**Affiliations:** 1 Department of Frigid Zone Medicine, College of High Altitude Military Medicine, Army Medical University, Chongqing, China; 2 Key Laboratory of Extreme Environmental Medicine, Ministry of Education of China, Chongqing, China; 3 Department of Emergency Medicine, Shigatse Branch, Xinqiao Hospital, Third Military Medical University, Shigatse, China

**Keywords:** Hematological parameters, Acute mountain sickness, Uric acid, Albumin, Uric acid to albumin ratio

## Abstract

**Objective:**

To screen and identify pre-plateau hematological parameters that can predict the risk of acute mountain sickness (AMS) in individuals rapidly ascending to high altitudes.

**Methods:**

A total of 297 healthy adult males with an average age of 20.88 ± 1.76 years were included in the study. Baseline data were collected at an altitude of 300 m using questionnaire surveys, on-site testing, and hematological parameter tests (including routine blood and serum biochemical parameters with validated clinical detection systems). After ascending to an altitude of 4,200 m, the Lake Louise Score (LLS) 2018 and physiological indicator measurements were obtained on the second day. AMS was defined as an LLS 2018 ≥3 points accompanied by headache. A univariate logistic regression model was used to screen for the hematological parameters associated with AMS. Multivariate logistic regression was then used to adjust for covariates and determine the relationship between pre-plateau albumin (ALB) levels, uric acid (UA) levels, UA-to-ALB ratio (UAR), and AMS risk. Additionally, the predictive value was assessed using the receiver operating characteristic (ROC) curve, and the optimal cutoff value was determined using the Youden index. Finally, a subgroup analysis was conducted to determine the consistency of associations.

**Results:**

The incidence of AMS after arriving at the 4,200 m plateau was 29.97%. Univariate logistic regression analysis revealed associations between factors at 300 m, including heart rate, alcohol consumption, ALB, UA, and AMS. In the fully adjusted multivariate Model 3, pre-plateau ALB, UA, and UAR showed moderate predictive performance for AMS, with area under the ROC curve values of 0.710, 0.701, and 0.711, respectively (all *P* < 0.001). The optimal cutoff values were 47.25 g/L for ALB, 420.10 μmol/L for UA, and 7.45 for UAR. The UAR exhibited favorable predictive performance in populations with a body mass index <25 kg/m^2^, a history of low-altitude residence, or alcohol consumption.

**Conclusion:**

Pre-plateau UA levels, ALB levels, and UAR are independent predictors of AMS risk in young healthy Chinese males. All three biomarkers exhibited moderate predictive performance, with the UAR showed particular utility in specific subgroups.

## Introduction

Acute mountain sickness (AMS) is a common health problem in individuals who rapidly ascend to altitudes above 2,500 m (Luks, Swenson & Bärtsch, 2017). Its incidence is closely correlated with the severity of hypoxia and increases markedly with altitude, ranging from 7% at 2,200 m to >52% at 4,559 m (Gatterer et al., 2024). Typical symptoms include headache, gastrointestinal symptoms, fatigue/weakness, and dizziness/light-headedness (Gatterer et al., 2024). If not treated properly, AMS can progress to high-altitude pulmonary or cerebral edema, leading to life-threatening complications (Burtscher et al., 2023). Recently, an increasing number of people have relocated to high altitude for various purposes, such as tourism and work. Concerns regarding the potential health impact of AMS can impose many physical and mental burdens. Therefore, it is necessary to assess and prevent AMS effectively before reaching high altitude.

Numerous epidemiological and clinical studies have identified a wide range of AMS risk factors categorized as environmental and individual determinants (Gatterer et al., 2024). AMS incidence increases in a dose-dependent manner with altitude and ascent rate, with rapid ascent being the most important modifiable risk factor. Elevated body mass index (BMI), excessive alcohol consumption, tobacco consumption, and inadequate pre-acclimatization are well-established individual predictors (Gatterer et al., 2024; Burtscher et al., 2023, 2026). Nevertheless, these traditional factors have critical limitations for clinical risk stratification: they are largely demographic or behavioral characteristics, explain only a small fraction of the marked interindividual variability in AMS susceptibility and offer no mechanistic insights into the pathophysiological processes underlying AMS pathogenesis (Sibomana et al., 2021; Wang et al., 2024a). This underscores the urgent need for objective pre-plateau biomarkers to reliably predict individual AMS risks and enable targeted prevention strategies. In this study, the “plateau” refers to the Qinghai-Tibet Plateau, with a pre-plateau baseline established in Chongqing (~300 m) and a high-altitude study site located at 4,200 m. This marked altitudinal gradient offers a natural experimental framework to identify pre-plateau biomarkers predictive of individual AMS susceptibility.

Blood biomarkers represent a promising solution to address this unmet clinical need, as they are objective, quantifiable, and can be measured before high-altitude exposure to identify at-risk individuals (Gatterer et al., 2024). Accumulating evidence has demonstrated that the pathogenesis of AMS involves complex interactions between hypoxic stress, oxidative stress, systemic inflammation, and fluid homeostasis regulation (Pena et al., 2022), all of which can be reflected by circulating blood parameters. Routine hematological and biochemical indicators, including serum albumin (ALB), uric acid (UA), and other markers of systemic nutritional status and inflammatory response, are widely used to assess general physiological conditions and may detect metabolic abnormalities associated with hypoxic adaptation (Lin et al., 2023; Guo et al., 2023).

Preliminary studies have explored the predictive value of baseline hematological indicators for AMS, representing a promising alternative to conventional demographic and lifestyle risk factors (Wang et al., 2018). A prospective cohort study of 46 participants found that pre-plateau serum ALB levels were significantly lower in individuals who did not develop AMS than in those who did, further indicating that elevated plasma ALB levels may have a predictive value for AMS (Ye et al., 2023). In addition to albumin, UA and platelet indices have attracted research attention. Another small-sample case-control study demonstrated that pre-plateau serum UA and platelet distribution width (PDW) were significantly higher in AMS-resistant individuals than in their AMS-susceptible counterparts, suggesting their potential predictive value (Liu et al., 2023). However, these early studies had important limitations: they were constrained by limited sample sizes, which resulted in inadequate statistical power and restricted population generalizability. Most importantly, they focused almost exclusively on individual biomarkers rather than composite indices.

The UA-to-ALB ratio (UAR) is a composite biomarker that integrates information from both UA and ALB. It has previously been shown to reflect the inflammatory and oxidative stress status more comprehensively than either marker alone (Wang, Sun & Ren, 2024). It was initially used to evaluate cardiovascular diseases and is now widely applied to predict acute kidney injury, peripheral artery disease, and other pathological conditions (Wang, Sun & Ren, 2024; Özgür et al., 2021; Oflar et al., 2023). Given the established roles of inflammation and oxidative stress in the pathogenesis of AMS and the promising preliminary findings for UA and ALB individually, it is reasonable to hypothesize that UAR may have a superior predictive performance compared with its individual components.

However, existing studies are limited by small sample sizes and a lack of direct comparisons between markers. Therefore, large prospective cohorts are required to validate these potential predictors. This large-scale prospective cohort study investigated the predictive value of pre-plateau UA, ALB, and UAR for AMS in healthy young males and compared their relative efficiencies. Our findings aimed to provide convenient hematological indicators for pre-plateau AMS risk stratification and support early targeted prevention.

## Methods

### Participants and design

This study was conducted in Chongqing, China in May 2023. A total of 316 participants were recruited for the study. All participants relocated to high altitude regions for occupational reasons, including personnel undertaking military duties, Tibet-aid staff, infrastructure construction workers, and other plateau-based working professionals. The inclusion criteria were: (1) healthy males aged 18–25 years; (2) no chronic cardiopulmonary, hepatorenal, hematological, or metabolic diseases (including hypertension, diabetes, and gout); (3) no acute infection, trauma, or surgery within the past 2 weeks; and (4) signed written informed consent. Exclusion criteria were as follows: history of high-altitude exposure (altitude ≥2,500 m) within the past 2 years, including both short-term travel and permanent residence. A total of 19 participants were excluded based on the following criteria: 14 had incomplete baseline data, and five were lost to follow-up after entering high-altitude areas (unable to be reached for the post-plateau evaluation).

All participants traveled as a single group by train from Chongqing (300 m, pre-plateau baseline altitude) to Gyangze County, Shigatse City, Tibet (4,200 m, plateau) over a 3-day period. Baseline data, including questionnaire surveys, on-site testing, and hematological parameter tests (routine blood tests and serum biochemical analyses), were collected in the morning in Chongqing prior to departure. After arrival at the plateau study site, the 2018 Lake Louise Score (LLS 2018) questionnaire was administered, and physiological parameters were measured within 48 h (Fig. 1) (Roach et al., 2018). To minimize confounding factors, participants were permitted *ad libitum* access to their usual meals and drinking water, whereas smoking, alcohol consumption, tea, and all caffeinated beverages were strictly prohibited throughout the study period.

**Figure 1:**
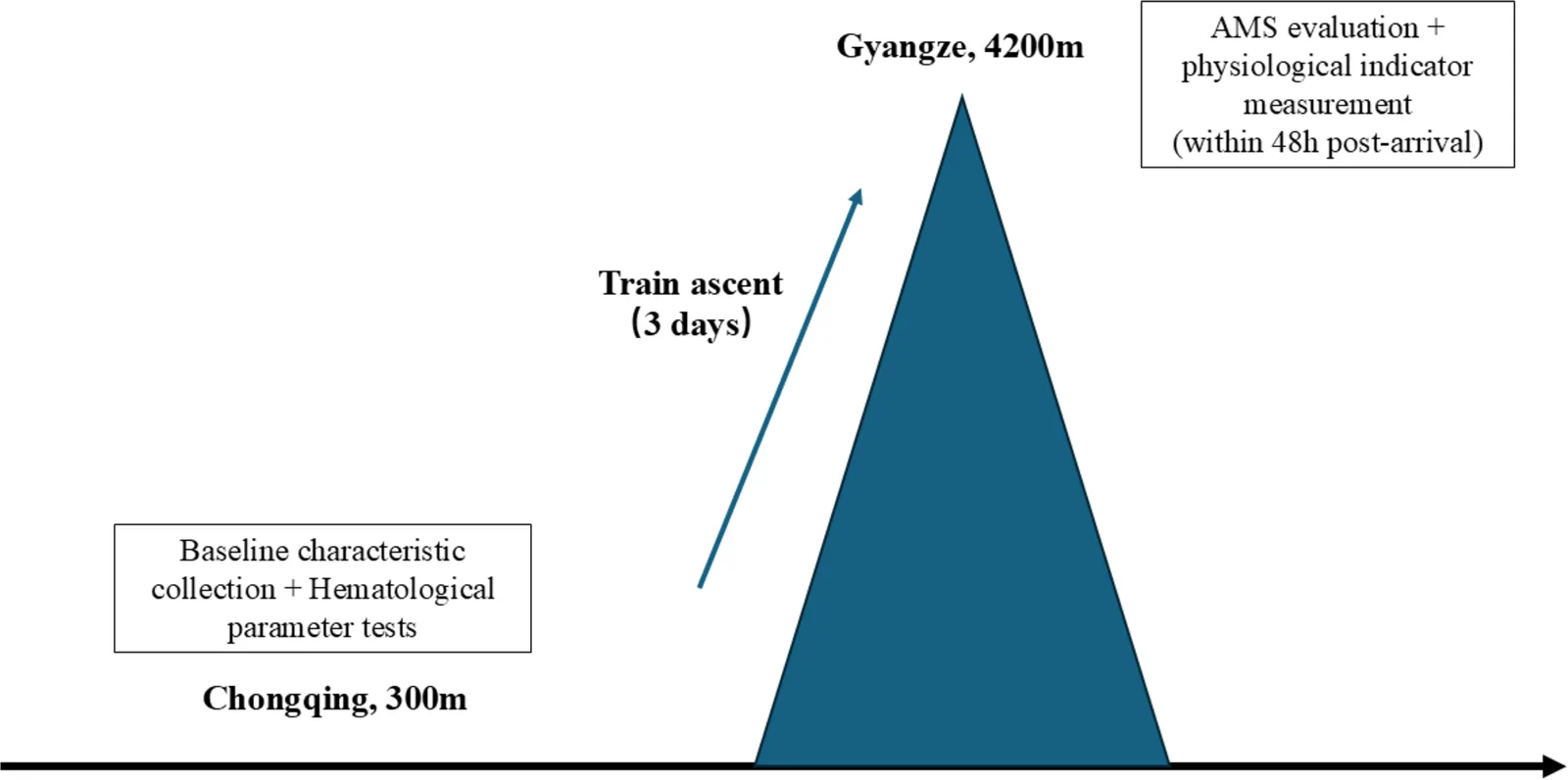
Schematic illustration of the study design.

All the study procedures were conducted in strict accordance with the Declaration of Helsinki (ethical principles for medical research involving human subjects). Prior to enrollment, all participants received standardized verbal and written explanations of the study protocol, were fully informed of the potential risks and benefits, and provided written informed consent. Ethical approval was obtained from the Army Medical University Ethics Committee (Approval Identifier 2022 No. 20-02).

### Baseline characteristic collection

Baseline characteristics were collected through face-to-face questionnaire surveys and on-site testing. Self-administered health questionnaires captured physiological indicators (age, height, weight, heart rate (HR), and blood pressure (systolic blood pressure, SBP; diastolic blood pressure, DBP)), lifestyle factors (tobacco smoking, alcohol consumption, tea consumption, coffee consumption, and beverage consumption), education level (below college and college and above), and history of high-altitude residence (altitude <1,000 m and altitude ≥1,000 m). Trained researchers measured resting HR, SBP, and DBP using a calibrated electronic sphygmomanometer YE670A (Jiangsu Yuyue Medical Equipment & Supply Co., Ltd., Danyang, China) after 10 min of supine rest. Regarding lifestyle factors, for tobacco smoking, the option “yes” was defined as smoking at least one cigarette per day for a continuous period of ≥6 months. For alcohol, tea, coffee, and beverage consumption, the option “yes” was defined as consuming these at least once per week for a continuous period of ≥6 months (World Health Organization, 1997; Wang et al., 2023; An, Kim & Seo, 2023).

### Whole blood collection and hematological parameter tests

All hematological assessments and serum biochemical analyses were conducted using fasting venous blood samples. Trained clinical nurses performed antecubital venipuncture on each participant using standard disposable vacuum blood collection devices. Specimens were collected in two types of Becton, Dickinson and Company (BD) Vacutainer tubes: ethylenediaminetetraacetic acid (EDTA) anticoagulant tubes (purple cap) for hematological parameter measurements and serum separator tubes (SST, yellow cap) for biochemical analysis. All specimens were immediately placed in portable cold boxes, stored temporarily at 2–8 °C on-site, and transported to the Clinical Laboratory of Southwest Hospital, Army Medical University for analysis. The following parameters were measured for each participant using clinically validated detection systems: white blood cell count (WBC), red blood cell count (RBC), hemoglobin (HGB), hematocrit (HCT), mean corpuscular volume (MCV), mean corpuscular hemoglobin (MCH), mean corpuscular Hb concentration (MCHC), platelet count (PLT), neutrophil count (NEU), lymphocyte count (LYMPH), monocyte count (MON), eosinophil count (EOS), basophil count (BAS), coefficient of variation of RDW (RDW-CV), standard deviation of RDW (RDW-SD), mean platelet volume (MPV), PDW, plateletcrit (PCT), platelet-to-larger cell ratio (P_LCR), total protein (TP), albumin (ALB), globulin (GLB), alanine aminotransferase (ALT), aspartate aminotransferase (AST), γ-glutamyl transpeptidase (GCT), alkaline phosphatase (ALP), total bilirubin (TBIL), direct BIL (DBIL), indirect BIL (IBIL), total bile acid (TBA), total cholesterol (TCH), triglycerides (TG), high-density lipoprotein cholesterol (HDLC), low-density lipoprotein cholesterol (LDLC), UA, urea (UN), creatinine (CR), and glucose (GLU).

### AMS evaluation and physiological indicator measurement

The severity of AMS was determined by the result of the LLS 2018 questionnaire, which included various symptoms (headache, gastrointestinal symptoms, fatigue/weakness, and dizziness) rated on a scale of 0 to 3. AMS was defined as a Lake Louise AMS total score of ≥3 points, accompanied by headache (Roach et al., 2018), which is the most widely used standard in AMS epidemiological studies and recommended by the 2018 LLS consensus, ensuring the comparability of our results with those of the previous studies.

Peripheral oxygen saturation (SpO_2_) and HR were measured using a validated finger pulse oximeter YX310 (Jiangsu Yuyue Medical Equipment & Supply Co., Ltd., Danyang, China) in a quiet room, consistent with the timing of the AMS evaluation.

### Statistical analysis

Statistical analyses were performed using IBM SPSS 21.0 Statistics (IBM Corp., Armonk, NY, USA). Statistical significance was set at *P* < 0.05. Quantitative variables are expressed as mean values ± standard deviation, and qualitative variables are reported as numbers (percentages). Independent-sample *t*-tests were used to compare continuous baseline characteristics and hematological parameters (including UA and ALB) between the AMS and non-AMS groups. Chi-square tests were used to compare categorical variables between the two groups.

Univariate logistic regression analysis was performed using AMS as the dependent variable to screen for potential predictive factors and to evaluate the association between each candidate variable and AMS development. Odds ratio (OR) was defined as the ratio of the odds of AMS among individuals with a higher biomarker level relative to those with a lower level. An OR > 1 suggested an elevated risk of AMS, while an OR < 1 indicated a reduced risk. This analysis preliminarily confirmed the significant correlations among UA, ALB, and AMS. Variables with *P* < 0.05 in independent samples *t*-tests and chi-square tests were selected as covariates for subsequent multivariate analyses. Multivariate logistic regression models were constructed to assess the independent effects of UA, ALB, and UAR on AMS risk, after adjusting for confounding factors. Model 1 was adjusted for age and BMI; Model 2 was adjusted for age, BMI, HR, high altitude, and alcohol and coffee consumption; and Model 3 was adjusted for age, BMI, HR, high altitude, alcohol consumption, coffee consumption, PLT, BAS, PDW, and DBIL.

The predictive performance of UAR for AMS was evaluated using receiver operating characteristic (ROC) curve analysis. The area under the curve (AUC) with a 95% confidence interval (CI) was calculated, and the optimal cutoff value was determined using the Youden index. The AUC ranges from 0 to 1, where a larger value suggests better overall performance of a predictive test (Park, Goo & Jo, 2004). In general, an AUC of 0.5 suggests no discrimination (*i.e*., ability to predict or diagnose patients with and without the disease or condition based on the test), 0.7 to 0.8 is considered acceptable, 0.8 to 0.9 is considered excellent, and more than 0.9 is considered outstanding (Mandrekar, 2010). Subgroup analyses were conducted to examine the influence of lifestyle factors, education level, and high-altitude residence on AMS incidence. Clinically relevant cutoff values were chosen *a priori* to ensure comparability with the existing literature (World Health Organization, 1997; Wang et al., 2023; An, Kim & Seo, 2023; World Health Organization, 2000). Finally, only BMI (<25 or ≥25 kg/m^2^), history of high-altitude residence (altitude <1,000 m or altitude ≥1,000 m), and alcohol consumption (yes or no) showed statistically significant differences.

## Results

### Baseline characteristics of participants

The baseline characteristics of the participants prior to the 4,200 m ascent are shown in Table 1. The mean age was 20.88 ± 1.76 years. Individuals with BMI <25 kg/m^2^ accounted for 88.9% of the cohort. The mean HR, SBP, and DBP were 63.9 ± 7.1 beats/min, 120.9 ± 8.3 mmHg, and 75.5 ± 8.5 mmHg, respectively.

**Table 1:** Characteristic of participants in the non-AMS and AMS groups.

	Total	Non-AMS	AMS	*P*-value
	*N* = 297	*N* = 208	*N* = 89	
Age (years)	20.88 ± 1.76	20.88 ± 1.84	20.90 ± 1.56	0.274
HR (beats/min)	63.9 ± 7.1	63.3 ± 6.9	65.6 ± 7.4	0.567
SBP (mmHg)	120.9 ± 8.3	121.1 ± 8.5	120.4 ± 7.9	0.316
DBP (mmHg)	75.5 ± 8.5	75.6 ± 8.6	75.3 ± 8.2	0.501
BMI (kg/m^2^)				0.841
<25	264 (88.9%)	184 (88.5%)	80 (89.9%)	
≥25	33 (11.1%)	24 (11.5%)	9 (10.1%)	
History of high-altitude residence				0.888
<1,000	213 (71.7%)	150 (72.1%)	63 (70.8%)	
≥1,000	84 (28.3%)	58 (27.9%)	26 (29.2%)	
Education level				0.080
Below college	46 (15.5%)	27 (13.0%)	19 (21.3%)	
College or above	251 (84.5%)	181 (87.0%)	70 (78.7%)	
Tobacco smoking				1.000
None	144 (48.5%)	101 (48.6%)	43 (48.3%)	
Yes	153 (51.5%)	107 (51.4%)	46 (51.7%)	
Alcohol consumption				0.012*
None	189 (64.3%)	142 (68.9%)	47 (53.4%)	
Yes	105 (35.7%)	64 (31.1%)	41 (46.6%)	
Tea consumption				0.627
None	276 (92.9%)	192 (92.3%)	84 (94.4%)	
Yes	21 (7.1%)	16 (7.7%)	5 (5.6%)	
Coffee consumption				0.009*
None	287 (96.6%)	205 (98.6%)	82 (92.1%)	
Yes	10 (3.4%)	3 (1.4%)	7 (7.9%)	
Beverage consumption				0.067
None	34 (11.4%)	28 (13.5%)	6 (6.7%)	
Yes	263 (88.6%)	180 (86.5%)	83 (93.3%)	
ALB (g/L)	47.27 ± 1.78	47.12 ± 1.84	47.62 ± 1.57	0.208
UA (μmol/L)	363.72 ± 57.13	369.27 ± 60.10	350.75 ± 47.31	0.043*
Plateau-HR (beats/min)	88.4 ± 9.7	87.4 ± 9.4	90.74 ± 10.0	0.000*
Plateau-SpO_2_ (%)	86.4 ± 4.3	87.0 ± 4.2	85.1 ± 4.2	0.000*

**Notes:**

AMS, acute mountain sickness; HR, heart rate at resting; SBP, systolic blood pressure; DBP, systolic blood pressure; BMI, body mass index; ALB, albumin; UA, uric acid.

* *P* value indicates *P* < 0.05.

### Characteristics of the non-AMS and AMS groups

According to the LLS score after reaching the plateau, the incidence of AMS was 29.97% in the study. The AMS group had a significantly lower mean plateau-SpO_2_ and a significantly higher mean plateau-HR than the non-AMS group. The hematological parameters collected at 300 m in the AMS and non-AMS groups are summarized in Table S1. PLT, BAS, PDW, DBIL, and UA levels were significantly different between the AMS and non-AMS groups. UA levels in the non-AMS group were 369.27 ± 60.10 μmol/L and 350.75 ± 47.31 μmol/L in the AMS group.

### Univariate analysis

Univariate logistic regression was performed for all hematological parameters tested at 300 m to identify the susceptibility factors for AMS. Using LLS as a dependent variable, univariate logistic regression analysis showed that HR (OR, 1.047; 95% CI [1.010–1.085]; *P* = 0.012), alcohol consumption (OR, 0.517; 95% CI [0.310–0.862]; *P* = 0.012), and coffee consumption (OR, 0.171; 95% CI [0.043–0.679]; *P* = 0.012) were associated with AMS (Table S2). Among the hematological parameters, ALB (OR, 1.175; 95% CI [1.019–1.355]) and UA (OR, 0.994; 95% CI [0.989–0.999]) levels were significantly associated with AMS (Table S3).

### Correlation analysis

Multivariate logistic regression was performed for parameters that showed a significant association with AMS in univariate logistic regression and baseline characteristics or showed significant differences between the non-AMS and AMS groups. Analysis of the association between ALB levels and AMS risk revealed a positive correlation among the three models. Specifically, in Model 1, the OR was 1.167, with 95% CI ranging from 1.012 to 1.345. In Model 2, the OR increased to 1.189 and the 95% CI was [1.023–1.383]. In Model 3, the OR further rose to 1.207, with a 95% CI of [1.033–1.411]. These results suggest that high ALB levels are associated with an increased AMS risk.

In contrast, UA exhibited a significant protective effect against AMS. In Model 1, the OR was 0.994, with a 95% CI of [0.989–0.999]. In Model 2, the OR remained at 0.994, and the 95% CI was [0.989–0.999]. In Model 3, the OR increased slightly to 0.995, with a 95% CI of [0.990–1.000]. This finding indicates that higher UA levels may be associated with a reduced AMS risk. With regard to the UAR, the results of Model 1 (OR, 0.721; 95% CI [0.577–0.900]), Model 2 (OR, 0.730; 95% CI [0.578–0.922]), and Model 3 (OR, 0.741; 95% CI [0.584–0.941]) showed that the UAR was a protective factor against AMS (Table 2).

**Table 2:** Relationships between pre-plateau ALB, UA and UAR and AMS risk.

Variable		OR	95% CI	β	*P*-value
ALB	Crude	1.175	[1.019–1.355]	0.161	0.026*
	Model 1	1.167	[1.012–1.345]	0.155	0.034*
	Model 2	1.189	[1.023–1.383]	0.173	0.024*
	Model 3	1.207	[1.033–1.411]	0.188	0.018*
UA	Crude	0.994	[0.989–0.999]	−0.006	0.011*
	Model 1	0.994	[0.989–0.999]	−0.006	0.013*
	Model 2	0.994	[0.989–0.999]	−0.006	0.026*
	Model 3	0.995	[0.990–1.000]	−0.005	0.044*
UAR	Crude	0.719	[0.576–0.897]	−0.330	0.003*
	Model 1	0.721	[0.577–0.900]	−0.328	0.004*
	Model 2	0.730	[0.578–0.922]	−0.314	0.008*
	Model 3	0.741	[0.584–0.941]	−0.299	0.014*

**Notes:**

ALB, albumin; UA, uric acid; UAR UA-to-ALB ratio. Model 1 was adjusted for age and BMI; Model 2 was adjusted for age, BMI, HR, History of high-altitude residence, Alcohol consumption, Coffee consumption; Model 3 was adjusted for age, BMI, HR, History of high-altitude residence, Alcohol consumption, Coffee consumption, PLT, BAS, PDW, DBIL.

* *P* value indicates *P* < 0.05.

### ROC curve analysis

ROC curve was used to evaluate the predictive effects of pre-plateau ALB, UA, and the UAR for AMS. As presented in Fig. 2, the AUC of the UA, ALB, and UAR predictions in Model 3 were 0.701 (95% CI [0.636–0.765]), 0.710 (95% CI [0.658–0.772]), and 0.711 (95% CI [0.648–0.774]), respectively. The optimal cutoff values were 47.25 g/L for ALB, 420.10 μmol/L for UA, and 7.45 for UAR. At these thresholds, UAR achieved the highest Youden index of 0.351, corresponding to a sensitivity of 73.6% and specificity of 61.6%; ALB exhibited the highest sensitivity (79.3%) among the three markers, whereas UA demonstrated a specificity of 61.1% (Table S4).

**Figure 2:**
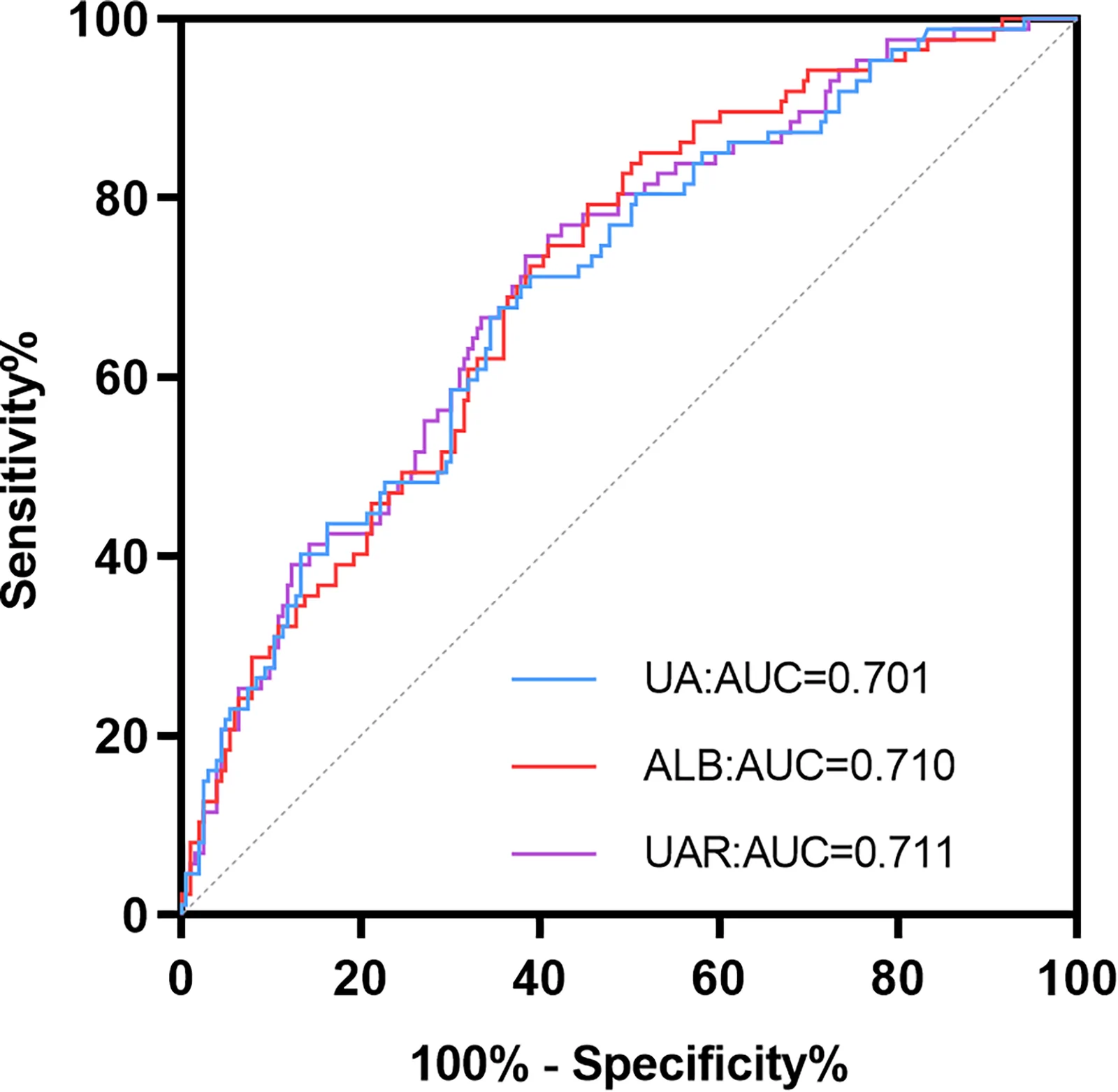
ROC curve analysis of ALB, UA, UAR to discriminate AMS individuals from non-AMS ones.

### Subgroup analysis

We conducted a subgroup analysis based on the BMI, history of residential altitude, and alcohol consumption (Fig. 3). The results showed a significant association between UAR and AMS risk among participants with a BMI <25 kg/m^2^ (OR, 0.754; 95% CI [0.582–0.978]), a history of low-altitude residence (OR, 0.708; 95% CI [0.543–0.922]), or alcohol consumption (OR, 0.607; 95% CI [0.399–0.923]). In addition, the AUC of UAR were both 0.701 for participants with BMI <25 kg/m^2^ and BMI ≥25 kg/m^2^. The AUC of the UAR in participants with a history of low- and high-altitude residence were 0.716 and 0.694, respectively. The AUC of the UAR in participants who did not consume alcohol and those who did were 0.686 and 0.713, respectively (Fig. S1).

**Figure 3:**
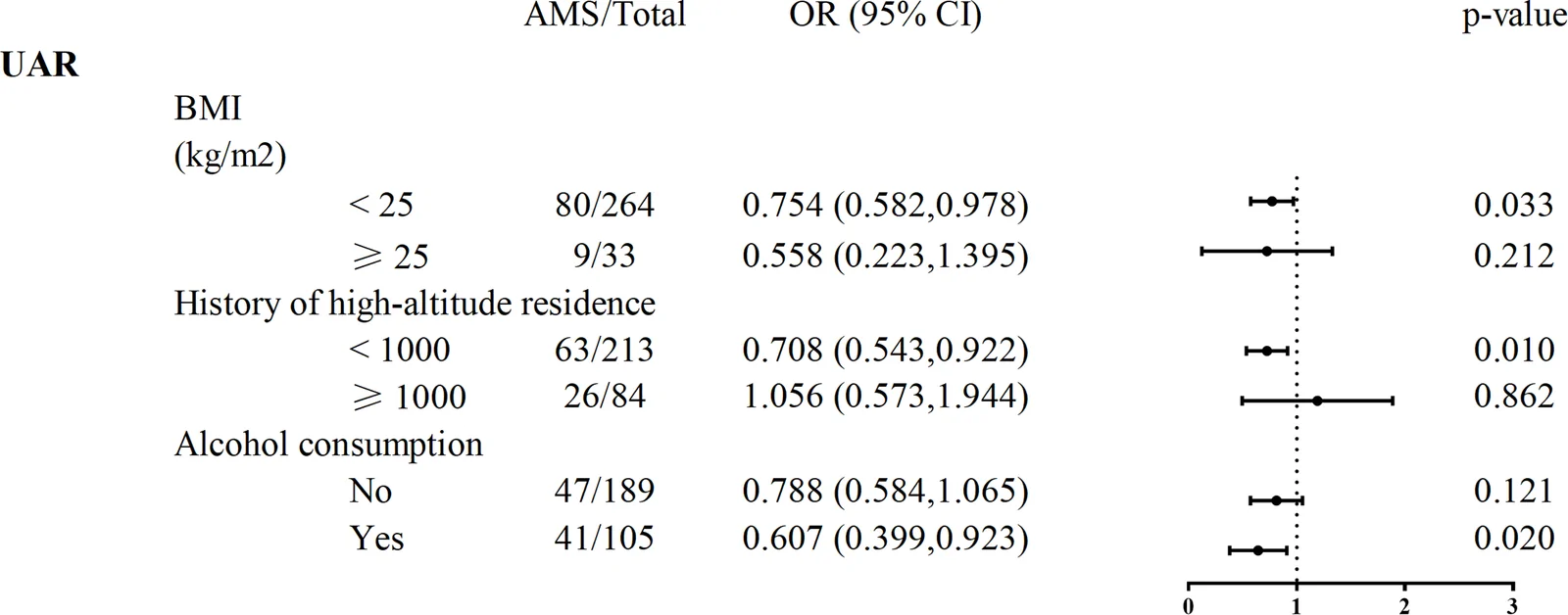
Odds ratio for the AMS in subgroups.

## Discussion

As human activity at high altitude continues to expand, the ability to predict individual susceptibility to AMS has become increasingly important. Identifying susceptible individuals and implementing appropriate preventive measures can efficiently decrease the risk of AMS, especially before ascent. In our study, incidence of AMS after reaching plateau (4,200 m) by train was 29.97%. Pre-plateau ALB and UA levels were associated with AMS risk and had a predictive value for susceptible individuals. Furthermore, ROC curve analysis showed that UAR exhibited comparable predictive performance to UA and ALB levels alone in predicting AMS. ALB had the highest sensitivity but the lowest specificity, while UA and UAR showed balanced diagnostic performance, with UAR having a slightly higher Youden index. Finally, the subgroup analysis showed that UAR had a favorable predictive value in populations with BMI <25 kg/m^2^, a history of low-altitude residence, or alcohol consumption.

The incidence and severity of AMS depend on the rate of ascent and altitude reached, length of time at altitude, degree of physical exertion, and an individual’s physiological susceptibility (Gatterer et al., 2024). In our prospective cohort, the overall incidence of AMS was 29.97%, which is lower than that reported in most previous studies (Luks, Swenson & Bärtsch, 2017; Gatterer et al., 2024; Burtscher et al., 2023; Burtscher et al., 2026). This reduced incidence can be largely explained by the gradual ascent protocol: participants traveled to high altitude *via* train over a 3-day period, allowing for partial physiological acclimatization during ascent. Furthermore, our study population was restricted to healthy young males aged 18–25 years with excellent physical fitness and no pre-existing medical conditions, which are known protective factors against AMS. Importantly, we observed significant differences in plateau-SpO₂ and plateau-HR between AMS and non-AMS individuals. These findings provide objective physiological evidence supporting the biological validity of the 2018 LLS for AMS classification, demonstrating that individuals meeting the clinical AMS criteria exhibit the expected pathophysiological responses to hypoxemia and sympathetically mediated tachycardia following acute hypoxic exposure.

ALB is the most abundant protein in human blood and is primarily involved in regulating plasma colloid osmotic pressure, maintaining the balance between intravascular and extravascular spaces, and ensuring normal blood volume (Xu et al., 2022). Previous research has indicated that an increase in plasma ALB levels following acute hypoxia exposure may have predictive value for AMS (Ye et al., 2023). In our study, we found that higher pre-plateau ALB levels were a risk factor for AMS. A recent large-scale longitudinal proteomic study involving 312 participants identified ALB as one of only 18 clinical biomarkers significantly associated with AMS susceptibility following rapid ascent to high altitude (Yang et al., 2022). Within the normal physiological range, higher pre-plateau ALB levels likely reflect altered hemorheological properties or endothelial dysfunction (Raberin et al., 2019). Three plausible mechanisms may explain this association: (1) relative contraction of baseline plasma volume, predisposing patients to excessive hemodynamic stress under hypoxia (Beidleman et al., 2016); (2) ALB-mediated independent regulation of inflammation and endothelial function, with higher baseline levels potentially indicating a preexisting pro-inflammatory/pro-oxidative state (Belinskaia, Jenkins & Goncharov, 2023); and (3) genetic polymorphisms affecting ALB metabolism or distribution (Zhang et al., 2014). Furthermore, elevated ALB concentrations may increase blood viscosity and impair microcirculatory perfusion and oxygen delivery under hypoxic conditions (Beidleman et al., 2016). The high sensitivity (79.3%) of ALB at a cutoff of 47.25 g/L makes it a valuable initial screening tool for identifying individuals at potential risk, although its relatively low specificity (54.7%) limits its utility as a stand-alone diagnostic marker. One limitation is the absence of dietary intake data, which influences normal-range ALB levels (Cederholm et al., 2019). Although pre-plateau ALB shows promise as an AMS predictor, the underlying mechanisms require further investigation.

UA is the end product of purine metabolism in the human body and plays dual physiological roles in oxidative stress and inflammatory responses (Zhu et al., 2024; Wang et al., 2024b). Within normal physiological range, UA acts as a potent endogenous antioxidant and anti-inflammatory agent, whereas when levels exceed the normal range (hyperuricemia), it transitions to a pro-oxidant and pro-inflammatory mediator that contributes to the pathogenesis of multiple diseases. Previous studies have shown that individuals resistant to AMS have significantly higher UA levels within the normal range than those susceptible to AMS (Liu et al., 2023). Recent studies suggest that UA plays a significant role in the pathogenesis of diseases, such as stroke, depression, epilepsy, and neurodegenerative diseases (Zhu et al., 2024; Wang et al., 2024b; Wan et al., 2024; Alrouji et al., 2024). In our study, we found that higher pre-plateau UA levels within the normal range served as a protective factor against AMS. AMS symptoms, such as headache, nausea, dizziness, and fatigue, are associated with hypoxia-induced increases in cerebral blood volume, inflammation, and trigeminovascular system activation (Gatterer et al., 2024). Within the normal range, UA plays a significant antioxidant role by scavenging peroxynitrite and hydroxyl radicals and protecting neurons and vascular endothelial cells during cerebral ischemia reperfusion (Leira et al., 2023). UA also exerts neuroprotective effects by inhibiting radical oxygen species accumulation, maintaining Ca^2+^ homeostasis, preserving mitochondrial function (Yu et al., 1998), promoting the elevation of brain-derived neurotrophic factor levels, and alleviating oxidative stress in ischemic brains (Cutler et al., 2019). Additionally, UA can mitigate neuroinflammation by inhibiting early activation of microglia through the Interleukin-6/Signal transducer and activator of transcription 3signaling pathway (Aliena-Valero et al., 2021). Collectively, UA may alleviate AMS symptoms through its antioxidant, anti-inflammatory, neuroprotective, and vascular endothelial-regulating effects. Consequently, higher pre-plateau UA levels within the normal range are predictive of AMS resistance.

Given the opposing predictive roles of ALB and UA, a composite index of UAR was developed, and its predictive value for AMS risk was further investigated. We found that UAR exhibited slight superiority in terms of the ROC value and demonstrated particular predictive advantages for populations with a history of low-altitude residence. This is the first study to analyze the relationship between UAR and AMS and demonstrates that UAR is a protective factor against AMS using a multimodel adjusted regression analysis. The nearly identical AUC values of the three biomarkers, combined with UAR’s slightly higher Youden index, indicated that integrating the opposing biological signals of these two biomarkers may provide complementary information to individual biomarkers for assessing hypoxic adaptation capacity. In some studies, the pathological elevation of UAR positively correlated with disease severity and was an independent risk factor for disease occurrence (Wang, Sun & Ren, 2024; Özgür et al., 2021; Oflar et al., 2023, 2024). In our study, UAR was considered a protective factor, possibly because the study population consisted of healthy young adult males with both UA and ALB levels within the normal physiological ranges. The UAR value in this study population was 7.71 ± 1.27 μmol/g, and the optimal cutoff value for AMS prediction determined by the Youden index method was 7.45 μmol/g. Notably, both the mean population value and the predictive cutoff fall entirely within the previously established normal UAR reference range of 7.20–8.38 μmol/g (Oflar et al., 2023; Zhang et al., 2024; Li et al., 2022), indicating that the predictive value of UAR for AMS stems from physiological fluctuations within the normal range rather than pathological deviations.

Subgroup and AUC analyses results indicated that UAR exhibited a significant protective effect against AMS in populations with a BMI <25 kg/m^2^, a history of low-altitude residence, or alcohol consumption. Previous studies have shown a correlation between BMI and AMS risk (Karinen et al., 2010; Hsu et al., 2015). In this study, most individuals (88.90%) had a normal BMI (<25 kg/m^2^); thus, the subgroup analysis was predominantly influenced by normal-weight individuals. Consequently, future investigations with purposive oversampling of overweight and obese individuals are warranted to further validate the predictive performance of UAR across diverse adiposity categories. The incidence of AMS was high in low-altitude residential populations. However, subgroup analyses based on history of altitude residence have not been conducted in previous risk prediction studies. Our study indicates that the UAR performs better in predicting AMS risk in participants with a history of low-altitude residence, providing novel insights into AMS prediction and prevention in specific populations. Regarding the impact of alcohol consumption on health, recent research suggests that alcohol intake is an important factor influencing AMS (Yang et al., 2020; Horiuchi, Mitsui & Uno, 2024). Subgroup analysis showed that the UAR had a better predictive performance for AMS in participants with alcohol consumption. One potential mechanism is that long-term alcohol consumption affects ALB synthesis and UA metabolism in the liver, leading to greater individual differences in ALB and UA levels in the alcohol-consuming population. The UAR can more stably reflect the systemic oxidative stress and inflammatory state of the alcohol consumption population by integrating changes in the two indicators, thus showing a more accurate predictive effect (Özgür et al., 2021). Alcohol consumption is an important factor affecting AMS susceptibility (Horiuchi, Mitsui & Uno, 2024), and the UAR can comprehensively reflect the metabolic changes caused by alcohol intake, which further improves its predictive efficiency. These data further confirm the predictive value of UAR for AMS risk, particularly its predictive advantage in specific populations.

This prospective cohort study had several strengths, including a low loss-to-follow-up rate and rigorous adjustments for covariates. However, several limitations should be acknowledged. First, the study population consisted exclusively of young healthy adult males, limiting generalizability to women or individuals with comorbidities. In addition, this research was conducted at a single center without external validation cohorts to verify our findings. Therefore, future multi-center studies recruiting more diverse populations are required. Second, all three biomarkers showed only moderate predictive performance, which is consistent with most published AMS prediction studies and reflects the multifactorial nature of AMS pathogenesis. Although these easily measurable biomarkers provide valuable references for risk stratification, they cannot replace comprehensive clinical assessment. Third, the specific pathological mechanisms through which UA and ALB affect AMS require further investigation. Subsequent studies should involve metabolomic analyses of blood samples from the population to identify the specific mechanisms of influence.

## Conclusion

This study found that the pre-plateau hematological parameters of UA, ALB, and UAR were associated with AMS risk. All three biomarkers exhibit comparable moderate predictive performance for AMS, with UAR demonstrating a favorable predictive value in specific subgroups.

## Supplemental Information

10.7717/peerj.21770/supp-1 Supplemental Information 1 ROC curve analysis of UAR to discriminate AMS individuals from non-AMS individuals across various subgroups.

10.7717/peerj.21770/supp-2 Supplemental Information 2 Hematologic parameters of participants in the AMS group and Non-AMS group. WBC white blood cell count, RBC red blood cell count, HGB hemoglobin, HCT hematocrit, MCV mean corpuscular volume, MCH mean corpuscular hemoglobin, MCHC mean corpuscular HGB concentration, PLT platelet count, NEU neutrophil count, LYMPH lymphocyte count, MON monocyte count, EOS eosinophil count, BAS basophil count, RDW-CV coefficient of variation of RDW, RDW-SD standard deviation of RDW, MPV mean platelet volume, PDW platelet distribution width, PCT plateletcrit, P_LCR platelet-to-larger cell ratio, TP total protein, ALB albumin, GLB globulin, ALT alanine aminotransferase, AST aspartate aminotransferase, GCT γ-glutamyl transpeptidase, ALP alkaline phosphatase, TBIL total bilirubin, DBIL direct bilirubin, IBIL indirect bilirubin, TBA total bile acid, TCH total cholesterol, TG triglycerides, HDLC high-density lipoprotein cholesterol, LDLC low-density lipoprotein cholesterol, UA uric Acid, UN urea, CR creatinine, GLU glucose. *p value represents *p* < 0.05.

10.7717/peerj.21770/supp-3 Supplemental Information 3 Baseline variables in the logistic regression equation. HR heart rate at resting, SBP systolic blood pressure, DBP systolic blood pressure, BMI body mass index. *p value represents p < 0.05.

10.7717/peerj.21770/supp-4 Supplemental Information 4 Hematologic variables in the logistic regression equation. WBC white blood cell count, RBC red blood cell count, HGB hemoglobin, HCT hematocrit, MCV mean corpuscular volume, MCH hemoglobin, MCHC mean corpuscular HGB concentration, PLT platelet count, NEU neutrophil count, LYMPH lymphocyte count, MON monocyte count, EOS eosinophil count, BAS basophil count, RDW-CV coefficient of variation of RDW, RDW-SD standard deviation of RDW, MPV mean platelet volume, PDW platelet distribution width, PCT plateletcrit, P_LCR platelet-to-larger cell ratio, TP total protein, ALB albumin, GLB globulin, ALT alanine aminotransferase, AST aspartate aminotransferase, GCT γ-glutamyl transpeptidase, ALP alkaline phosphatase, TBIL total bilirubin, DBIL direct bilirubin, IBIL indirect bilirubin, TBA total bile acid, TCH total cholesterol, TG triglycerides, HDLC high-density lipoprotein cholesterol, LDLC low-density lipoprotein cholesterol, UA uric Acid, UN urea, CR creatinine, GLU glucose. *P value represents *P* < 0.05.

10.7717/peerj.21770/supp-5 Supplemental Information 5 Predictive performance of pre-plateau UA, ALB and UAR for AMS.

10.7717/peerj.21770/supp-6 Supplemental Information 6 Description and classification of potential covariates.

10.7717/peerj.21770/supp-7 Supplemental Information 7 Raw data.

10.7717/peerj.21770/supp-8 Supplemental Information 8 Categorical Data. The description and classification of potential covariates

10.7717/peerj.21770/supp-9 Supplemental Information 9 2018 Lake Louise Acute Mountain Sickness (AMS) Score questionnaire.

10.7717/peerj.21770/supp-10 Supplemental Information 10 STROBE Checklist.

## Additional Information and Declarations

### Data Availability

The following information was supplied regarding data availability:

The raw measurements are available in the Supplemental Files.
