# Supplementary material for: Prediction of acute mountain sickness risk by pre-plateau uric acid and albumin levels in Chinese young males: a prospective cohort study
Source: PeerJ. 2026 Oct 14;14:e21770. doi: 10.7717/peerj.21770 (PMC13676272; doi:10.7717/peerj.21770)
Supplement: Supplementary file 1 [file peerj-14-21770-s005.docx]

Supplementary Table 4 Predictive performance of pre-plateau UA, ALB and UAR for AMS

| Variable | AUC | 95% CI | P value | Optimal cutoff | Sensitivity (%) | Specificity (%) | Youden Index |
| --- | --- | --- | --- | --- | --- | --- | --- |
| ALB (g/L) | 0.710 | 0.648,0.772 | 0.000 | 47.25 | 79.3 | 54.7 | 0.340 |
| UA(μmol/L) | 0.701 | 0.636,0.765 | 0.000 | 420.10 | 71.3 | 61.1 | 0.323 |
| UAR | 0.711 | 0.648,0.774 | 0.000 | 7.45 | 73.6 | 61.6 | 0.351 |
