# Supplementary material for: Prediction of acute mountain sickness risk by pre-plateau uric acid and albumin levels in Chinese young males: a prospective cohort study
Source: PeerJ. 2026 Oct 14;14:e21770. doi: 10.7717/peerj.21770 (PMC13676272; doi:10.7717/peerj.21770)
Supplement: Supplementary file 2 [file peerj-14-21770-s006.docx]

**Supplementary Table 5.** Description and classification of potential covariates

| **Potential covariates** | **Description** | **Classification** |
| --- | --- | --- |
| Age |  |  |
| BMI | Overweight and obesity were defined as a BMI greater than or equal to 25 kg/m2 | < 25  ≥ 25 |
| History of high-altitude residence | Geographically, plateau is defined as an area above 1000 m above sea level. | <1000 or ≥1000 m |
| Educational level |  | Below college, college or above |
| Smoking | " Smoking " were defined as individuals smoking at least one cigarette daily for at least six months | Yes or No |
| Drinking | " Drinking " were defined as individuals drinking at least one cup of alcohol daily for at least six months | Yes or No |
| Tea | " Tea " were defined as individuals drink tea at least one cup of tea daily for at least six months | Yes or No |
| Coffee | " Coffee " were defined as individuals drink coffee at least one cup of coffee daily for at least six months | Yes or No |
| Beverage | " Beverage " were defined as individuals drink beverage at least one cup of beverage daily for at least six months | Yes or No |
