# Supplementary material for: Prediction of acute mountain sickness risk by pre-plateau uric acid and albumin levels in Chinese young males: a prospective cohort study
Source: PeerJ. 2026 Oct 14;14:e21770. doi: 10.7717/peerj.21770 (PMC13676272; doi:10.7717/peerj.21770)
Supplement: Supplementary file 4 [file peerj-14-21770-s009.docx]

**Table S2.** 2018 Lake Louise Acute Mountain Sickness Score

Headache

0—None at all

1—A mild headache

2—Moderate headache

3—Severe headache, incapacitating

Gastrointestinal symptoms

0—Good appetite

1—Poor appetite or nausea

2—Moderate nausea or vomiting

3—Severe nausea and vomiting, incapacitating

Fatigue and/or weakness

0—Not tired or weak

1—Mild fatigue/weakness

2—Moderate fatigue/weakness

3—Severe fatigue/weakness, incapacitating

Dizziness/light-headedness

0—No dizziness/light-headedness

1—Mild dizziness/light-headedness

2—Moderate dizziness/light-headedness

3—Severe dizziness/light-headedness, incapacitating

This Lake Louise AMS score is for use by investigators studying AMS.

1. The Lake Louise AMS score is designed as a self-report questionnaire that research volunteers complete on their own.

2. The Lake Louise AMS score for an individual is the sum of the score for the four symptoms (headache, nausea/vomiting, fatigue, and dizziness/light-headedness). For a positive AMS definition, it is mandatory to have a headache score of at least one point, and a total score of at least three points.

Reference

Roach RC, Hackett PH, Oelz O, et al. The 2018 Lake Louise Acute Mountain Sickness Score. *High Alt Med Biol*. 2018;19(1):4-6. doi:10.1089/ham.2017.0164
