# Supplementary material for: Prediction of acute mountain sickness risk by pre-plateau uric acid and albumin levels in Chinese young males: a prospective cohort study
Source: PeerJ. 2026 Oct 14;14:e21770. doi: 10.7717/peerj.21770 (PMC13676272; doi:10.7717/peerj.21770)
Supplement: Supplementary file 6 [file peerj-14-21770-s002.doc]

**Supplementary Table 1** Hematologic parameters of participants in the AMS group and Non-AMS group

|  | Total | Non-AMS | AMS | P-value |
| --- | --- | --- | --- | --- |
|  | N = 297 | N = 208 | N = 89 |  |
| WBC (10^9^/L) | 6.00 ± 0.31 | 5.97 ±1.29 | 6.06 ±1.40 | 0.310 |
| RBC (10^12^/L) | 5.04 ± 0.31 | 5.03 ±0.33 | 5.06 ± 0.28 | 0.218 |
| HGB (g/L) | 153.29 ± 7.88 | 152.79 ±7.70 | 154.46 ± 8.22 | 0.648 |
| HCT (%) | 48.79 ± 2.12 | 45.69 ±2.08 | 46.02 ± 2.20 | 0.507 |
| MCV (fL) | 90.94 ± 4.56 | 90.88 ± 4.74 | 91.06 ± 4.11 | 0.806 |
| MCH (pg) | 30.47 ± 1.72 | 30.42 ± 1.74 | 30.59 ± 1.66 | 0.778 |
| MCHC (g/L) | 335.04 ± 7.12 | 334.72 ± 6.78 | 335.80 ± 7.84 | 0.173 |
| PLT (10^9^/L) | 235.60 ± 48.61 | 237.31 ± 52.37 | 231.62 ± 38.42 | 0.028* |
| NEU (10^9^/L) | 3.44 ± 1.15 | 3.44 ± 1.12 | 3.44 ± 1.22 | 0.311 |
| LYMPH (10^9^/L) | 1.97 ± 0.43 | 1.95 ± 0.42 | 2.03 ± 0.47 | 0.123 |
| MON (10^9^/L) | 0.37 ± 0.12 | 0.37 ± 0.12 | 0.37 ± 0.13 | 0.657 |
| EOS (10^9^/L) | 0.18 ± 0.13 | 0.18 ± 0.12 | 0.19 ± 0.14 | 0.199 |
| BAS (10^9^/L) | 0.03 ± 0.02 | 0.03 ± 0.19 | 0.03 ± 0.14 | 0.028* |
| NEU (%) | 56.01 ± 8.67 | 56.48 ± 8.26 | 54.89 ± 9.53 | 0.270 |
| LYMPH (%) | 33.78 ± 7.33 | 33.48 ± 7.01 | 34.49 ± 8.03 | 0.149 |
| MON (%) | 6.37 ± 2.42 | 6.41 ± 2.66 | 6.27 ± 1.75 | 0.742 |
| EOS (%) | 3.00 ± 2.04 | 2.99 ± 2.01 | 3.02 ± 2.13 | 0.525 |
| BAS (%) | 0.54 ± 0.28 | 0.56 ± 0.30 | 0.51 ± 0.24 | 0.654 |
| RDW-CV (%) | 13.00 ± 0.59 | 13.01 ± 0.59 | 12.98 ± 0.59 | 0.569 |
| RDW-SD (%) | 42.17 ± 1.96 | 42.13 ± 1.98 | 42.26 ± 1.93 | 0.577 |
| MPV (fL) | 10.60 ± 1.05 | 10.55± 1.08 | 10.73 ± 0.99 | 0.195 |
| PDW (%) | 15.27 ± 2.48 | 15.33 ± 1.63 | 15.14 ± 3.79 | 0.005* |
| PCT (%) | 0.25 ± 0.04 | 0.25 ± 0.04 | 0.25 ± 0.040 | 0.271 |
| P_LCR (%) | 30.02 ± 7.24 | 29.71 ± 7.36 | 30.76 ± 6.94 | 0.351 |
| TP (g/L) | 76.39 ± 5.24 | 76.40 ± 3.41 | 76.36 ± 8.09 | 0.166 |
| ALB (g/L) | 47.27 ± 1.78 | 47.12 ± 1.84 | 47.62 ± 1.57 | 0.208 |
| G (g/L) | 29.37 ± 2.91 | 29.31 ± 3.00 | 29.52 ± 2.69 | 0.098 |
| ALT (IU/L) | 20.43 ± 9.46 | 20.69 ±10.01 | 19.83 ±8.04 | 0.173 |
| AST (IU/L) | 22.12 ± 7.09 | 22.16 ± 6.57 | 22.03 ± 8.22 | 0.777 |
| GCT (IU/L) | 21.73 ± 11.93 | 21.91 ± 13.49 | 21.32 ± 7.14 | 0.078 |
| ALP (IU/L) | 111.00 ± 29.81 | 110.27 ± 31.33 | 112.69 ± 26.01 | 0.125 |
| TBIL (μmol/L) | 15.33 ± 6.65 | 14.87 ± 6.37 | 16.40 ± 7.18 | 0.051 |
| DBIL (μmol/L) | 3.02 ± 1.16 | 2.95 ± 1.09 | 3.21 ± 1.29 | 0.046* |
| IBIL (μmol/L) | 12.31 ± 5.56 | 11.93 ± 5.36 | 13.19 ± 5.95 | 0.066 |
| TBA (μmol/L) | 3.26 ± 3.86 | 3.31 ±4.22 | 3.14 ± 2.88 | 0.704 |
| TCH (mmol/L) | 3.94 ± 0.65 | 3.92 ± 0.66 | 3.99 ± 0.62 | 0.669 |
| TG (mmol/L) | 0.85 ± 0.41 | 0.87 ± 0.39 | 0.80 ± 0.43 | 0.385 |
| HDLC (mmol/L) | 1.20 ± 0.20 | 1.19 ± 0.20 | 1.23 ± 0.21 | 0.799 |
| LDLC (mmol/L) | 2.38 ± 0.47 | 2.36 ± 0.48 | 2.42 ± 0.45 | 0.399 |
| UA (μmol/L) | 363.72 ± 57.13 | 369.27 ± 60.10 | 350.75 ± 47.31 | 0.043* |
| UN (mmol/L) | 5.12 ± 2.42 | 4.99 ± 1.15 | 5.42 ± 4.05 | 0.106 |
| CR (μmol/L) | 74.38 ± 8.57 | 74.92 ± 8.67 | 73.10 ± 8.22 | 0.681 |
| GLU (μmol/L) | 4.35 ± 0.36 | 4.37 ± 0.38 | 4.29 ± 0.31 | 0.105 |

WBC white blood cell count, RBC red blood cell count, HGB hemoglobin, HCT hematocrit, MCV mean corpuscular volume, MCH mean corpuscular hemoglobin, MCHC mean corpuscular HGB concentration, PLT platelet count, NEU neutrophil count, LYMPH lymphocyte count, MON monocyte count, EOS eosinophil count, BAS basophil count, RDW-CV coefficient of variation of RDW, RDW-SD standard deviation of RDW, MPV mean platelet volume, PDW platelet distribution width, PCT plateletcrit, P_LCR platelet-to-larger cell ratio, TP total protein, ALB albumin, GLB globulin, ALT alanine aminotransferase, AST aspartate aminotransferase, GCT γ-glutamyl transpeptidase, ALP alkaline phosphatase, TBIL total bilirubin, DBIL direct bilirubin, IBIL indirect bilirubin, TBA total bile acid, TCH total cholesterol, TG triglycerides, HDLC high-density lipoprotein cholesterol, LDLC low-density lipoprotein cholesterol, UA uric Acid, UN urea, CR creatinine, GLU glucose.

*p value indicates p < 0.05.

**Supplementary Table 2** Baseline variables in the logistic regression equation

| Variable | OR | 95% CI | P value |
| --- | --- | --- | --- |
| Age | 1.005 | 0.872,1.158 | 0.947 |
| HR (beats/min) | 1.047 | 1.010,1.085 | 0.012* |
| SBP (mmHg) | 0.990 | 0.961,1.020 | 0.505 |
| DBP (mmHg) | 0.996 | 0.967,1.026 | 0.779 |
| BMI (kg/m^2^) | 1.159 | 0.516,2.606 | 0.720 |
| Highland | 0.937 | 0.542,1.621 | 0.816 |
| Education | 1.820 | 0.951,3.480 | 0.070 |
| Smoking | 0.990 | 0.603,1.627 | 0.969 |
| Drinking | 0.517 | 0.310,0.862 | 0.012* |
| Tea | 1.400 | 0.497,3.947 | 0.525 |
| Coffee | 0.171 | 0.043,0.679 | 0.012* |
| Beverage | 0.465 | 0.185,1.165 | 0.102 |

**Supplementary Table 3.** Clinical blood variables in the logistic regression equation

| Variable | OR | 95% CI | P value |
| --- | --- | --- | --- |
| WBC (10^9^/L) | 1.047 | 0.870,1.261 | 0.627 |
| RBC (10^12^/L) | 1.262 | 0.575,2.771 | 0.562 |
| HGB (g/L) | 1.028 | 0.995,1.062 | 0.096 |
| HCT (%) | 1.079 | 0.958,1.216 | 0.076 |
| MCV (fL) | 1.009 | 0.954,1.067 | 0.754 |
| MCH (pg) | 1.062 | 0.910,1.238 | 0.448 |
| MCHC (g/L) | 1.021 | 0.986,1.058 | 0.233 |
| PLT (10^9^/L) | 0.998 | 0.992,1.003 | 0.356 |
| NEU (10^9^/L) | 1.001 | 0.806,1.243 | 0.994 |
| LYMPH (10^9^/L) | 1.479 | 0.835,2.620 | 0.180 |
| MON (10^9^/L) | 1.218 | 0.151,9.831 | 0.853 |
| EOS (10^9^/L) | 1.517 | 0.221,10.412 | 0.671 |
| BAS (10^9^/L) | 0.000 | 0.000,121.630 | 0.179 |
| NEU (%) | 0.979 | 0.952,1.008 | 0.152 |
| LYMPH (%) | 1.019 | 0.985,1.055 | 0.274 |
| MON (%) | 0.974 | 0.867,1.093 | 0.650 |
| EOS (%) | 1.007 | 0.892,1.136 | 0.912 |
| BAS (%) | 0.548 | 0.214,1.407 | 0.211 |
| CV (%) | 0.915 | 0.596,1.407 | 0.687 |
| SD (%) | 1.033 | 0.910,1.173 | 0.618 |
| MPV (fL) | 1.179 | 0.929,1.495 | 0.175 |
| PDW (%) | 0.965 | 0.861,1.081 | 0.539 |
| PCT (%) | 0.840 | 0.002,362.465 | 0.955 |
| P_LCR (%) | 1.020 | 0.986,1.056 | 0.252 |
| TP (g/L) | 0.998 | 0.953,1.046 | 0.944 |
| ALB (g/L) | 1.175 | 1.019,1.355 | 0.026* |
| G (g/L) | 1.025 | 0.941,1.117 | 0.565 |
| ALT (IU/L) | 0.990 | 0.963,1.018 | 0.476 |
| AST (IU/L) | 0.997 | 0.962,1.034 | 0.880 |
| GCT (IU/L) | 0.996 | 0.974,1.018 | 0.699 |
| ALP (IU/L) | 1.003 | 0.994,1.011 | 0.523 |
| TBIL (μmol/L) | 1.034 | 0.997,1.072 | 0.071 |
| DBIL (μmol/L) | 1.209 | 0.979,1.492 | 0.078 |
| IBIL (μmol/L) | 1.040 | 0.996,1.086 | 0.077 |
| TBA (μmol/L) | 0.988 | 0.922,1.058 | 0.723 |
| TCH (mmol/L) | 1.182 | 0.805,1.733 | 0.394 |
| TG (mmol/L) | 0.630 | 0.306,1.298 | 0.210 |
| HDLC (mmol/L) | 2.784 | 0.838,9.248 | 0.095 |
| LDLC (mmol/L) | 1.277 | 0.754,2.165 | 0.363 |
| UA (μmol/L) | 0.994 | 0.989,0.999 | 0.011* |
| UN (mmol/L) | 1.076 | 0.948,1.222 | 0.257 |
| CR (μmol/L) | 0.976 | 0.946,1.004 | 0.094 |
| GLU (μmol/L) | 0.551 | 0.274,1.108 | 0.095 |
