# Supplementary material for: Prediction of acute mountain sickness risk by pre-plateau uric acid and albumin levels in Chinese young males: a prospective cohort study
Source: PeerJ. 2026 Oct 14;14:e21770. doi: 10.7717/peerj.21770 (PMC13676272; doi:10.7717/peerj.21770)
Supplement: Supplementary file 7 [file peerj-14-21770-s003.doc]

**Supplementary table 2** Baseline variables in the logistic regression equation

| Variable | OR | 95% CI | P value |
| --- | --- | --- | --- |
| Age | 1.005 | 0.872,1.158 | 0.947 |
| HR (beats/min) | 1.047 | 1.010,1.085 | 0.012* |
| SBP (mmHg) | 0.990 | 0.961,1.020 | 0.505 |
| DBP (mmHg) | 0.996 | 0.967,1.026 | 0.779 |
| BMI (kg/m^2^) | 1.159 | 0.516,2.606 | 0.720 |
| Highland | 0.937 | 0.542,1.621 | 0.816 |
| Education | 1.820 | 0.951,3.480 | 0.070 |
| Smoking | 0.990 | 0.603,1.627 | 0.969 |
| Drinking | 0.517 | 0.310,0.862 | 0.012* |
| Tea | 1.400 | 0.497,3.947 | 0.525 |
| Coffee | 0.171 | 0.043,0.679 | 0.012* |
| Beverage | 0.465 | 0.185,1.165 | 0.102 |

HR heart rate at resting, SBP systolic blood pressure, DBP systolic blood pressure, BMI body mass index.

*p value indicates p < 0.05.
